# Sparse regression models for unraveling group and individual associations in eQTL mapping

**DOI:** 10.1186/s12859-016-0986-9

**Published:** 2016-03-22

**Authors:** Wei Cheng, Yu Shi, Xiang Zhang, Wei Wang

**Affiliations:** Department of Computer Science, UNC at Chapel Hill, 201 S Columbia St., Chapel Hill, NC 27599 USA; Computer Science at the University of Illinois at Urbana-Champaign, 201 North Goodwin Avenue, Urbana, IL 61801 USA; Department of Elect. Eng. and Computer Science, Case Western Reserve University, 10900 Euclid Avenue, Cleveland, OH 44106 USA; Department of Computer Science, University of California, Los Angeles, 3531-G Boelter Hall, Los Angeles, CA 90095 USA

**Keywords:** eQTL mapping, Group-wise association, Computation efficiency

## Abstract

**Background:**

As a promising tool for dissecting the genetic basis of common diseases, expression quantitative trait loci (eQTL) study has attracted increasing research interest. Traditional eQTL methods focus on testing the associations between individual single-nucleotide polymorphisms (SNPs) and gene expression traits. A major drawback of this approach is that it cannot model the joint effect of a set of SNPs on a set of genes, which may correspond to biological pathways.

**Results:**

To alleviate this limitation, in this paper, we propose *geQTL*, a sparse regression method that can detect both group-wise and individual associations between SNPs and expression traits. *geQTL* can also correct the effects of potential confounders. Our method employs computationally efficient technique, thus it is able to fulfill large scale studies. Moreover, our method can automatically infer the proper number of group-wise associations. We perform extensive experiments on both simulated datasets and yeast datasets to demonstrate the effectiveness and efficiency of the proposed method. The results show that *geQTL* can effectively detect both individual and group-wise signals and outperforms the state-of-the-arts by a large margin.

**Conclusions:**

This paper well illustrates that decoupling individual and group-wise associations for association mapping is able to improve eQTL mapping accuracy, and inferring individual and group-wise associations.

**Electronic supplementary material:**

The online version of this article (doi:10.1186/s12859-016-0986-9) contains supplementary material, which is available to authorized users.

## Background

Expression quantitative trait loci (eQTL) mapping aims at identifying single nucleotide polymorphisms (SNPs) that influence the expression level of genes. It has been widely applied to analyze the genetic basis of gene expression and molecular mechanisms underlying complex traits [1, 2]. In a typical eQTL study, the association between each expression trait and each SNP is assessed separately [3–5]. This approach does not consider the interactions among SNPs and among genes. However, multiple SNPs may interact with each other and jointly influence the phenotypes [6]. This assumption will inevitably miss complex cases where multiple genetic variants jointly affect the co-expressions of multiple genes. It has been observed in biological experiments that the joint effect of multiple SNPs to a phenotype may be non-additive [6], and genes from the same biological pathway are usually co-regulated [7] by the same genetic basis. The biological process contains both individual effects and joint effects between SNPs and genes [8]. A straightforward approach to detect associations between sets of SNPs and a gene expression level can be done using the standard gene set enrichment analysis [9]. Wu et al. [10] further proposed the variance component models for SNP set testing. Braun et al. employed aggregation-based approaches to cluster SNPs [11]. In [12], Listgarten et al. further considered the potential confounding factors.

**Fig. 1:**
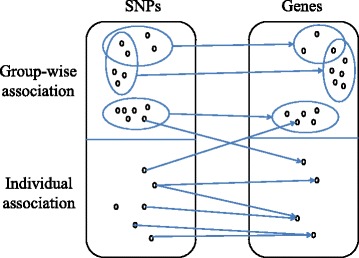
An illustration of individual and group-wise associations. Ellipses represent the groups of SNPs and genes. Blue arrows between SNPs and genes represent identified associations

However, there are two limitations for these approaches. First, these methods typically only consider SNPs from pre-defined pathways or gene ontology categories, which are far from being complete. Second, these methods can only detect the mapping of SNP set and a single gene expression level. To better elucidate the genetic basis of gene expression, it is a crucial challenge to understand how multiple modestly-associated SNPs interact to influence the a group of genes [6]. In this paper, we refer to this kind of eQTL mapping to find associations between group of SNPs and group of gene expression levels as the *group-wise* eQTL mapping. An example is shown in Fig. 1. Note that an ideal model should allow overlaps between SNP sets and between gene sets, that is, a SNP or gene may participate in multiple individual and group-wise associations [6]. In literature, *group-wise* eQTL mapping has attracted increasing research interest recently. For example, Xu et al. [13] proposed a two-graph-guided multi-task Lasso approach to infer group-wise eQTL mapping. However, it required the grouping information of both SNPs and genes available as prior knowledge, which may not be practical for many applications. Besides, it is not able to correct the effects of confounding factors.

In this paper, we propose a novel method, *geQTL*, to automatically detect individual and group-wise associations in eQTL studies. It uses a two-layer feature selection strategy and adopts efficient optimization techniques, which make it suitable for large scale studies. Moreover, geQTL can automatically infer the optimal number of group-wise associations. We perform extensive experiments on both simulated datasets and yeast datasets to demonstrate the effectiveness and efficiency of the proposed method.

## Methods

### Preliminaries

Important notations used in this paper are listed in Table 1. In this paper, for each sample, the data of SNPs and genes are denoted by column vectors. Let **x**=[*x*_1_,*x*_2_,…,*x*_*K*_]^T^ denote the *K* SNPs. Here, *x*_*i*_∈{0,1,2} denotes a random variable corresponding to the *i*-th SNP (For example, 0, 1, 2 may encode the homozygous major allele, heterozygous allele, and homozygous minor allele, respectively.). Let **z**=[*z*_1_,*z*_2_,…,*z*_*N*_]^T^ denote the *N* genes in the study. *z*_*j*_ denotes a continuous random variable corresponding to the *j*-th gene expression. Let $\mathbf {X}=\{{\mathbf {x}}_{h}|1\leq h\leq H\} \in {\mathbb {R}}^{K \times H}$ be the SNP matrix. We use $\mathbf {Z}=\{{\mathbf {z}}_{h}|1\leq h \leq H\}\in {\mathbb {R}}^{N\times H}$ to denote the matrix of gene expression levels. *H* denotes the number of samples in consideration.

**Table 1:** Notations

Symbols	Description
*K*	Number of SNPs
*N*	Number of genes
*H*	Number of samples
*M*	Number of group-wise associations
**x**	Random variables of *K* SNPs
**z**	Random variables of *N* genes
**y**	Latent variables to model group-wise associaiton
$\mathbf {X}\in {\mathbb {R}}^{K \times H}$	SNP matrix data
$\mathbf {Z} \in {\mathbb {R}}^{N \times H}$	Gene expression matrix data
$\mathbf {A} \in \mathbb {R}^{M\times K}$	Group-wise association coefficient matrix between **x** and **y**
$\mathbf {B} \in \mathbb {R}^{N\times M}$	Group-wise association coefficient matrix between **y** and **z**
$\mathbf {C} \in \mathbb {R}^{N\times K}$	Individual association coefficient matrix between **x** and **y**
*α*,*β*,*γ*,*ρ*	Regularization parameters
$\mathbf {R} \in \mathbb {R}^{N\times K}$	Indicator matrix showing which elements in **C** can be nonzero

Bold term means vector or matrix while non-bold term means scalar

The traditional linear regression model for association mapping between **x** and **z** is

(1) $$ \mathbf{z}=\mathbf{W}\mathbf{x}+\boldsymbol{\mu}+\mathbf{\epsilon},  $$

where **z** is a linear function of **x** with coefficient matrix **W**, ***μ*** is an *N*×1 translation factor vector. And **ε** is the additive noise of Gaussian distribution with zero-mean and variance *γ***I**, where *γ* is a scalar. That is, $\mathbf {\epsilon } \sim \mathcal {N}(\mathbf {0},\gamma \mathbf {I})$.

In association studies, sparsity is a reasonable assumption because only a small fraction of genetic variants are expected to be associated with a set of gene expression traits. This can be modeled as a feature selection problem. For example, the standard Lasso [5] can be used in association mapping, which applies *ℓ*_1_ penalty on **W** for sparsity.

If both **X** and **Z** are standardized, the objective function of Lasso is formulated as

(2) $$ \min_{\mathbf{W}} ||\mathbf{Z}-\mathbf{W}\mathbf{X}||_{F}^{2}+\eta||\mathbf{W}||_{1},  $$

where ||·||_*F*_ denotes the Frobenius norm, ||·||_1_ is the *ℓ*_1_-norm. *η* is the empirical parameter for the *ℓ*_1_ penalty. **W** is the parameter (also called weight) matrix parameterizing the space of linear functions mapping from **X** to **Z**.

Confounding factors, such as unobserved covariates, experimental artifacts and unknown environmental perturbations, may mask real signals and lead to spurious findings. LORS [14] uses a low-rank matrix $\mathbf {L}\in {\mathbb {R}}^{N\times H}$ to account for the variations caused by hidden factors. The objective function of LORS is

(3) $$ \min_{\mathbf{W},\mathbf{L}} ||\mathbf{Z}-\mathbf{W}\mathbf{X}-\mathbf{L}||_{F}^{2}+\eta||\mathbf{W}||_{1}+\rho ||\mathbf{L}||_{*},  $$

where ||·||_∗_ is the nuclear norm [14]. *ρ* is the regularization parameter to control the rank of **L**. **L** is a low-rank matrix assuming that there are only a small number of hidden factors influencing the gene expression levels.

When we fix {**W**, we can optimize {**L**} by using singular value decomposition (SVD) according to the following lemma.

#### **Lemma1**.

([15]) Suppose that matrix **O** has rank *r*. The solution to the optimization problem

(4) $$ \min_{\mathbf{S}}\frac{1}{2}||\mathbf{O}-\mathbf{S}||_{F}^{2}+\lambda ||\mathbf{S}||_{*}  $$

is given by $\mathbf {\widehat {S}}=\mathbf {H}_{\lambda }(\mathbf {O})$, where $\mathbf {H}_{\lambda }(\mathbf {O})=\mathbf {U}\mathbf {D}_{\lambda }\mathbf {V}^{\mathrm {T}}$ with **D**_*λ*_=diag[(*d*_1_−*λ*)_+_,…,(*d*_*r*_−*λ*)_+_], **UDV**^T^ is the Singular Value Decomposition (SVD) of **O**, **D**=diag[*d*_1_,…,*d*_*r*_], and $(d_{i}-\lambda)_{+}=\max ((d_{i}-\lambda),0), (1\leq i \leq r)$.

Thus, for fixed **W**, the formula for updating **L** is

(5) $$ \mathbf{L} \leftarrow \mathbf{H}_{\lambda}(\mathbf{Z}-\mathbf{\mathbf{W}\mathbf{X}})  $$

Both Lasso and LORS do not consider the existence of group-wise associations. Below, we will introduce the proposed model to infer both group-wise and individual associations for eQTL mapping.

### geQTL

In geQTL, individual associations between SNPs and genes are modeled by following the Lasso-based strategy. Group-wise associations are inferred using a two-layer feature selection method. Since multiple SNPs may have joint effect on a group of genes, and such effect may be accomplished through complex biological processes, we introduce latent variables to bridge sets of SNPs and sets of genes. Specifically, we assume that there exist latent factors regulating the gene expression level, which serve as bridges between the SNPs and the genes. The latent variables are denoted by **y**=[*y*_1_,*y*_2_,…,*y*_*M*_]^T^. Here, *M* (*M*≪*min*(*K*,*N*)) is the total number of latent variables representing group-wise associations. The relationship between **x** and **y** can be represented as

(6) $$ \mathbf{y} = \mathbf{Ax} + \boldsymbol{\epsilon}_{1},  $$

where

$$\boldsymbol{\epsilon}_{1} \sim \mathcal{N}\left(\mathbf{0}, {\sigma_{1}^{2}} \mathbf{I}_{M}\right). $$

$\mathbf {A} \in \mathbb {R}^{M\times K}$ denotes the matrix of coefficients between **x** and **y**. ${\sigma _{1}^{2}}\mathbf {I}_{M}$ denotes the variances of the additive noise. **I**_*M*_ is an identity matrix. Here we drop the intercept terms because the input data **X** and **Z** are normalized to zero mean and unit variance as preprocessing.

Similarly, the relationship between **y** and **z** can be represented as

(7) $$ \mathbf{z} = \mathbf{By} + \mathbf{Cx} + \boldsymbol{\epsilon}_{2},  $$

where

$$\boldsymbol{\epsilon}_{2} \sim \mathcal{N}\left(\mathbf{0}, {\sigma_{2}^{2}} \mathbf{I}_{N}\right). $$

$\mathbf {B} \in \mathbb {R}^{N\times M}$ denotes the matrix of coefficients between **y** and **z**, $\mathbf {C} \in \mathbb {R}^{N\times K}$ denotes the matrix of coefficients between **x** and **z** to encode the individual associations.

Note that Eq. (7) decouples the associations between SNPs and genes into two parts: one for individual associations represented as **Cx**, and another for group-wise associations represented as **By**. Next, we infer the group-wise associations by a two-layer feature selection strategy. We first remove the individual associations and denote

(8) $$ \tilde{\mathbf{Z}}=\mathbf{Z}- \mathbf{C}\mathbf{X}.  $$

Thus $\tilde {\mathbf {Z}}$ contains only group-wise effects. Next let

(9) $$ \mathbf{Y}=\mathbf{A}\mathbf{X}.  $$

Thus **Y** represents a low-rank transformation of the original SNP matrix. Each row of **Y** represents a group of SNPs. From Eq. (7), we have the following multiple-input-multiple-output (MIMO) linear system

(10) $$ \tilde{\mathbf{Z}}=\mathbf{B}\mathbf{Y}+\mathbf{E},  $$

where **E** is a Gaussian white-noise term. In Eq. (9) and (10), **A** and **B** should be sparse since a single gene is often influenced by a small number of SNPs and vice versa [12].

Therefore, the overall objective function is

(11) $$ \begin{aligned} \min_{\mathbf{A}, \mathbf{B}, \mathbf{C},\mathbf{L}} & loss(\mathbf{A}, \mathbf{B}, \mathbf{C},\mathbf{L})\\ & +\rho ||\mathbf{L}||_{*} + \alpha ||\mathbf{A}||_{1} + \beta ||\mathbf{B}||_{1} + \gamma ||\mathbf{C}||_{1}, \end{aligned}  $$

where *α*,*β*,*γ*,*ρ* are the regularization parameters, and the loss function is

(12) $$ loss(\mathbf{A}, \mathbf{B}, \mathbf{C},\mathbf{L}) = ||\mathbf{Z}-\mathbf{L}-(\mathbf{B} \mathbf{A} +\mathbf{C})\mathbf{X}||_{F}^{2}.  $$

Here, we choose different penalties for **A**,**B**,**C** because the sparsities of different matrices are typically of different scales.

### Optimization

The optimization for **L** can be achieved by following a similar approach as in [14]. To optimize **A**,**B**,**C**, many tools can be used to optimize the *ℓ*_1_ penalized objective function, e.g., the Orthant-Wise Limited-memory Quasi-Newton (OWL-QN) algorithm [16]. Due to space limitation, we omit the details. In the next, we devise optimization techniques that can dramatically improve the computational efficiency of geQTL.

### Boosting the computational efficiency

Given a large number of SNPs and gene expression traits, scalability of the algorithm is a crucial issue. We propose two improved models, geQTL ^+^ and geQTL-ridge, which optimize the search for significant individual associations, which is the main computational bottleneck of the algorithm.

#### geQTL ^+^

In a typical eQTL study, we usually have *M*≪*min*(*K*,*N*). Thus, the bottleneck of the algorithm is to optimize **C**. Our strategy is to confine the space of **C**. The intuition is that we only permit a small fraction of elements in **C** to be nonzero. It has been shown that if **Z** and **X** are standardized with zero mean and unit sum of squares, then **r**=*abs*(**ZX**^T^) is equal to the gene-SNP correlations (**r**_*gs*_=|*cor*(*z*_*g*_,*x*_*s*_)|) [17]. Since for many test statistics, e.g., *t*, *F*, *R*^2^, and LR, for the simple linear regression problem can be expressed as functions of the sample correlation **r**_*gs*_, e.g., *R*^2^=*r*^2^, and $t=\frac {r\sqrt {n-2}}{1-r^{2}}$, we can find a threshold according to the required *p*-value, such that test statistics exceeding the threshold are significant at the required significance level. The test statistics for every gene-SNP pair in **r** are compared with the threshold, and only those elements whose **r** are greater than the threshold are optimized. We denote $\mathbf {R} \in \mathbb {R}^{N\times K}$ as the indicator matrix indicating which elements in **C** can be nonzero (i.e., **r**_*gs*_>*threshold*).

#### geQTL-ridge

When *N* and *K* are extremely large, optimizing **C** may still be time-consuming, since it may take many iterations to converge with the *ℓ*_1_ constraint. Next, we introduce geQTL-ridge, which further improves the time efficiency with slight decrease in accuracy. The key idea is to use ridge regression for individual associations so that we can get a closed form solution for **C**. The objective function is shown in the following.

(13) $$ \begin{aligned} \min_{\mathbf{A}, \mathbf{B}, \mathbf{C},\mathbf{L}} & loss(\mathbf{A}, \mathbf{B}, \mathbf{C},\mathbf{L}) \\ & + \rho||\mathbf{L}||_{*}+ \alpha ||\mathbf{A}||_{1} + \beta ||\mathbf{B}||_{1} +\gamma||\mathbf{C}||_{2}^{2}, \\ & s.t. (\mathbf{C})_{i, j} ~is~nozero~only~if~(\mathbf{R})_{i, j} ~is~1. \end{aligned}  $$

##### **Theorem1**.

The solution of **C** in Eq. (13) is

(14) $$ \mathbf{c}_{i} \leftarrow \mathbf{d}_{i} \mathbf{X}^{\mathrm{T}} \mathbf{P}_{i} \left(\mathbf{P}_{i}^{\mathrm{T}} \mathbf{X} \mathbf{X}^{\mathrm{T}} \mathbf{P}_{i}+\gamma \mathbf{I}_{K}\right)^{-1} \mathbf{P}_{i}^{\mathrm{T}},  $$

where

$$\mathbf{c}_{i} = (\mathbf{C})_{i, :}, \mathbf{d}_{i} = (\mathbf{D})_{i, :}, $$

$$ \mathbf{D} = \mathbf{Z} -\mathbf{L} -\mathbf{B} \mathbf{A} \mathbf{X}, $$

and **P**_*i*_ is defined as in formula (19).

The proof of the Theorem 1 is in the following section.

### Proof of Theorem 1

#### *Proof*.

Recall that any ridge regression problem

(15) $$ \min_{\mathbf{a}} ||\mathbf{b} - \mathbf{a} \mathbf{Q}||_{2}^{2} + ||\mathbf{a} \mathbf{\Gamma}||_{2}^{2},  $$

where **a** is a row vector and **Q** has linearly independent rows, has the following solution

(16) $$ \mathbf{a} = \mathbf{b} \mathbf{Q}^{\mathrm{T}} (\mathbf{Q} \mathbf{Q}^{\mathrm{T}} + \mathbf{\Gamma} \mathbf{\Gamma}^{\mathrm{T}})^{-1}.  $$

Note that

(17) $$ loss(\mathbf{A}, \mathbf{B}, \mathbf{C},\mathbf{L}) = ||\mathbf{D} - \mathbf{C} \mathbf{X}||_{F}^{2} = \sum_{i=1}^{N} ||\mathbf{d}_{i} - \mathbf{c}_{i} \mathbf{X}||_{2}^{2},  $$

where **D**=**Z**−**L**−**BAX**, **c**_*i*_=(**C**)_*i*,:_ and **d**_*i*_=(**D**)_*i*,:_.

We have

(18) $$ \min_{\mathbf{C}} \quad loss(\mathbf{A}, \mathbf{B}, \mathbf{C},\mathbf{L}) = \sum_{i=1}^{N} \min_{\mathbf{c}_{i}} ||\mathbf{d}_{i} - \mathbf{c}_{i} \mathbf{X}||_{2}^{2},  $$

Taking into account that (**c**_*i*_)_*j*_ can be nonzero only if (**R**)_*i*,*j*_ is 1, we introduce **P**_*i*_, where **P**_*i*_ has *K* rows and $l_{i} = \sum _{j=1}^{K} (\mathbf {R})_{i,j}$ columns. And

(19) $$ (\mathbf{P}_{i})_{s, t} =\left\{ \begin{array}{ll} 1, & if~(\mathbf{R})_{i, s}~is~the~t\text{-}th~1~in~(\mathbf{R})_{i, :}; \\ 0, & otherwise. \end{array}\right.  $$

Then $\mathbf {c}_{i} = \mathbf {c}_{i} \mathbf {P}_{i} \mathbf {P}_{i}^{\mathrm {T}}$, $||\mathbf {d}_{i} - \mathbf {c}_{i} \mathbf {X}||_{2}^{2} +\gamma ||\mathbf {c}_{i}||_{2}^{2} = ||\mathbf {d}_{i} - (\mathbf {c}_{i} \mathbf {P}_{i}) \left (\mathbf {P}_{i}^{\mathrm {T}} \mathbf {X}\right)||_{2}^{2} +\gamma ||\mathbf {c}_{i} \mathbf {P}_{i}||_{2}^{2} $, and

(20) $$ \begin{aligned} \min_{\mathbf{c}_{i}} & ||\mathbf{d}_{i} - \mathbf{c}_{i} \mathbf{X}||_{2}^{2}+\gamma||\mathbf{c}_{i}||_{2}^{2},\\ s.t. & (\mathbf{c}_{i})_{j}~is~nozero~only~if~(\mathbf{R})_{i, j} ~is~ 1, \end{aligned}  $$

is solved by

(21) $$ \mathbf{c}_{i} = (\mathbf{c}_{i} \mathbf{P}_{i}) \mathbf{P}_{i}^{\mathrm{T}} = \mathbf{d}_{i} \mathbf{X}^{\mathrm{T}} \mathbf{P}_{i} \left(\mathbf{P}_{i}^{\mathrm{T}} \mathbf{X} \mathbf{X}^{\mathrm{T}} \mathbf{P}_{i}+\gamma \mathbf{I}_{K}\right)^{-1} \mathbf{P}_{i}^{\mathrm{T}}.  $$

Therefore,

$$\begin{array}{*{20}l} \min_{\mathbf{C}} \quad & loss(\mathbf{A}, \mathbf{B}, \mathbf{C},\mathbf{L})+\gamma||\mathbf{C}||_{2}^{2},\\ s.t. & (\mathbf{C})_{i, j}~is~nozero~only~if~(\mathbf{R})_{i, j} \textit{is} 1, \end{array} $$

is solved by $\mathbf {C} = \left (\mathbf {c}_{1}^{\mathrm {T}}, \ldots, \mathbf {c}_{N}^{\mathrm {T}}\right)^{\mathrm {T}},$ which leads to the update formula given in Eq. (14).

### Determining the number of hidden variables

In Eq. (12), we use **BA**+**C** to formulate the overall associations between SNPs and expression traits.

Two group-wise associations will not share the same group of SNPs (or genes), since otherwise these two group-wise associations can be combined into one. Therefore, every group-wise association should be unique and irreplaceable. Hence, following two conditions should be satisfied

- **A** has linearly independent rows. Since *M*≪*K*, this condition is equivalent to that **A** has full rank;
- **B** has linearly independent columns. Since *M*≪*N*, this condition is equivalent to that **B** has full rank.

When these two conditions are met, we have

(22) $$ M = \text{rank}(\mathbf{A}) = \text{rank}(\mathbf{B}) = \text{rank}(\mathbf{B} \mathbf{A}).  $$

The last equality holds because both **A** and **B** have full rank.

We have the following observation. The singular value decomposition (SVD) of **BA** has the form

$$ \mathbf{B} \mathbf{A} = \mathbf{U} \mathbf{\Sigma} \mathbf{V}^{\mathrm{T}}, $$

where **U** and **V** are unitary (orthogonal in our case) matrices, and **Σ** is a rectangular diagonal matrix with non-negative real numbers on the diagonal, which corresponds to singular values of **BA**. Since **U** and **V** are unitary and hence have full rank, we have

(23) $$  \begin{aligned} \text{rank}(\mathbf{B} \mathbf{A})\! &= \text{rank}\left(\mathbf{U} \mathbf{\Sigma} \mathbf{V}^{\mathrm{T}}\right) = \text{rank}(\mathbf{\Sigma}) \\ &= \text{the number of nonzero singular values of~} \mathbf{B} \mathbf{A}. \end{aligned}  $$

We compute **BA** by minimizing Eq. (12), which gives

(24) $$ \mathbf{B} \mathbf{A} = (\mathbf{Z} -\mathbf{L}- \mathbf{C} \mathbf{X}) \mathbf{X}^{\mathrm{T}}\left(\mathbf{X} \mathbf{X}^{\mathrm{T}}\right)^{-1}.  $$

Combine (22), (23), and (24), we find

(25) $$ \begin{aligned} M = \text{the number of nonzero singular values of }\\ \left(\mathbf{Z} -\mathbf{L}- \mathbf{C} \mathbf{X}\right) \mathbf{X}^{\mathrm{T}}\left(\mathbf{X} \mathbf{X}^{\mathrm{T}}\right)^{-1}. \end{aligned}  $$

Due to the existence of noise, we should allow small singular values to be considered as zero. Therefore, we can draw a plot with singular values of $(\mathbf {Z} -\mathbf {L}- \mathbf {C} \mathbf {X}) \mathbf {X}^{\mathrm {T}}\left (\mathbf {X} \mathbf {X}^{\mathrm {T}}\right)^{-1}$ in descending order and set *M* to be *k*, if the first *k* singular values are large and significantly greater than the (*k*+1)-th singular value.

Based on the discussion above, in order to find optimal *M*, we can first use Lasso to infer the initial value of **C**. Then, using Eq. 25, we can infer the optimal *M* at this stage. After that, we can optimize new **C**, and calculate new optimal *M*. We can repeat this procedure until *M* became stable or reach maximal number of iterations.

## Results

In this section, we perform extensive experimental study using both simulated and real eQTL datasets to evaluate the performance of our methods. For comparison, we select several state-of-the-art eQTL methods, including two-graph guided multi-task lasso (MTLasso2G) [13], FaST-LMM [12], SET-eQTL [18], LORS [14], Matrix eQTL [17] and Lasso [5]. Note that we did not compare with our previous work, GDL, in [19] because it needs to incorporate many prior knowledge, that is not relevant to this work. For all the methods, the tuning parameters are learned using cross validation. The discussion of setting proper number of group-wise associations *M* is included in the Additional file 1. The shrinkage of the coefficients is also presented in the Additional file 1.

### Simulated data

We use a similar setup for simulation study to that in [14]. First, 100 SNPs are randomly selected from the yeast eQTL dataset [20]. This gives birth to the matrix **X**. 100 gene expression profiles are generated by **Z**_*j*∗_=**β**_*j*∗_**X**+*Ξ*_*j*∗_+**E**_*j*∗_ (1≤*j*≤*N*), where $\mathbf {E}_{j*}\sim \mathcal {N}(0,\phi I)$ (*ϕ*=0.1) is used to simulate the Gaussian noise. To simulate the effects of confounding factors, we use *Ξ*_*j*∗_, drawn from $\mathcal {N}(\mathbf {0},\tau \Lambda)$. In this paper, we set *τ*=0.1. *Λ* is given by **FF**^T^. Here, $\mathbf {F}\in \mathbb {R}^{H\times J}$ and $\mathbf {F}_{ij}\sim \mathcal {N}(0,1)$. *J* denotes hidden factor number. In this paper, we set *J* to 10.

**Fig. 2:**
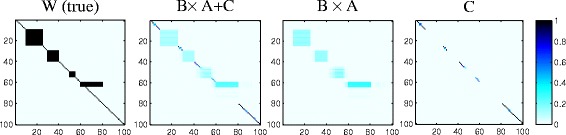
Ground truth of matrix W and associations estimated by geQTL. The x-axis represents SNPs and y-axis represents traits. Normalized absolute values of regression coefficients are used. Darker color implies stronger association. Number of group-wise associations *M* = 4

In the left most of Fig. 2, we illustrate **β**. Here, we set the association strength to 1. Totally, there exist four group-wise associations with different scales. The diagonal line represents the individual signals in *cis*-regulation.

In Fig. 2, we report the associations inferred by geQTL. Recall that group-wise associations can be inferred from matrix **A** and **B**, and individual associations can be inferred from matrix **C**. It is obvious that geQTL can detect both group-wise and individual signals.

**Fig. 3:**
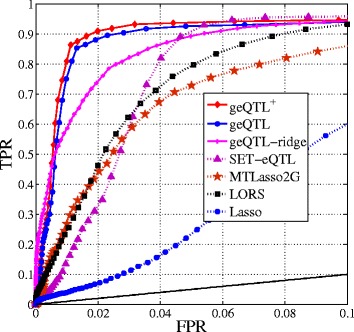
The ROC curve of FPR-TPR with different signal-to-noise ratios (*S* *N* *R*=0.13)

**Fig. 4:**
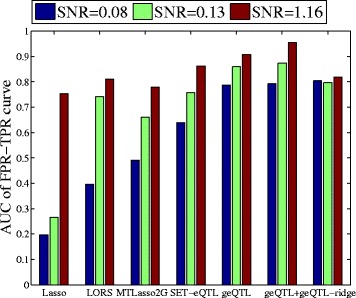
The AUCs curve

We use $SNR=\sqrt {\frac {Var(\mathbf {\beta }\mathbf {X})}{Var(\Xi + \mathbf {E})}}$ to denote the signal-to-noise ratio [14] in the eQTL datasets. Here, we fix *J*=10,*τ*=0.1. The *SNR*’s are controlled by using different *ϕ*’s. Using 50 simulated datasets with different *SNR*’s, we compare the proposed methods with the selected methods. Because FaST-LMM requires the input of genomic locations information (e.g., chromosome, base pair, etc), we will compare it on the real data set. The results are averaged over 50 different simulated datasets. **BA**+**C** is used to represent the association matrix in our method. Figure 3 shows the ROC curve of TPR-FPR (true positive rate - false positive rate) for performance comparison. Typically, we care more about the TPR when the FPR is small because it is important to evaluate the performance of model when controlling the maximum tolerated FPR. Thus, in Fig. 3, the ROC of interest for eQTL are generally shown in the range [0,0.1]. The corresponding areas under the TPR-FPR curve are shown in Fig. 4.

It can be seen that geQTL and geQTL ^+^ outperform all alternative methods by a large margin since they considers both individual and group-wise associations. We also observe that geQTL-ridge is not as good as geQTL and geQTL ^+^. This is because geQTL-ridge does not provide a sparse solution for individual associations. MTLasso2G is comparable to LORS. LORS can correct the effects of the confounders, however, it is not able to detect group-wise mappings. We also observe that by decoupling individual and group-wise associations, the proposed models (geQTL, geQTL ^+^, and geQTL-ridge) are more robust to noise than other methods.

### Yeast eQTL data

We also validated *geQTL* using the bench mark dataset–yeast (Saccharomyces cerevisiae) eQTL dataset. The dataset contains 112 yeast segregants generated from a cross of two inbred strains [20]. Originally, It contains 6229 gene epxressions and 2956 SNPs. SNPs with >10 *%* missing values in the remaining SNPs are imputed using the function fill.geno in R/qtl [21]. The neighboring SNPs with the same genotype profiles are combined, resulting in 1027 genotype profiles. Remove gene expression traits with missing values, we get 4474 expression profiles.

#### cis- and trans- analysis

We follow the standard *cis*-enrichment analysis that is used in [22, 23] for evaluation. Moreover, we use the *trans*-enrichment with a similar strategy [24]. Genes regulated by transcription factors (obtained from http://www.yeastract.com/download.php) are treated as *trans*-acting signals.

**Fig. 5:**
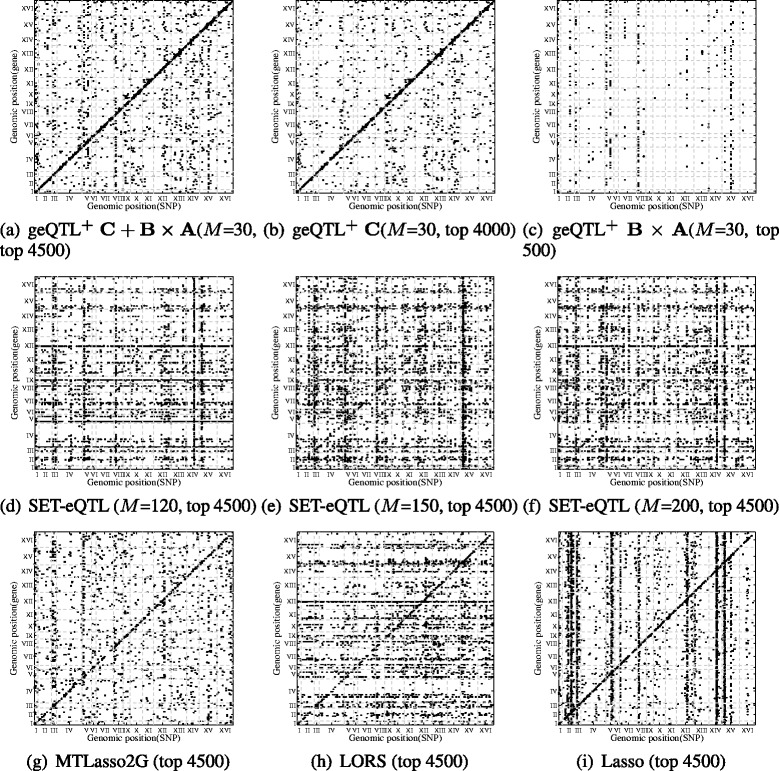
Significant associations reported on yeast eQTL dataset

In Table 2, we report the pairwise comparison using cis- and trans- enrichment analysis. We do not list geQTL separately from geQTL+, since geQTL+ is a faster version of geQTL. In this table, the methods are sorted (from top to bottom in the left column and from left to right in the top row) in decreasing order of performance. A *p*-value shows how significant a method on the left column outperforms a method in the top row in terms of *cis* and *trans* enrichments. We observe that geQTL ^+^ has significantly better *cis*-enrichment scores than the other models. For *trans*-enrichment, geQTL ^+^ is the best, and MTLasso2G comes in second, outperforming FaST-LMM, SET-eQTL, LORS, Matrix eQTL and Lasso. LORS outperforms Matrix eQTL and Lasso for both *cis*- and *trans*-enrichment. This is because LORS considers confounding factors while Matrix eQTL and Lasso does not. In total, these methods each detected about 6000 associations according to non-zero **W** values. We estimate FDR using 50 permutations as proposed in [14]. With FDR ≤ 0.01, geQTL ^+^ obtains about 4500 significant associations. The plots of all identified significant associations for different methods are given in Fig. 5. Obviously, we can see that **C**+**B**×**A** and **C** of geQTL ^+^ report weaker *trans*-regulatory bands while stronger *cis*-regulatory signals than other competitors.

**Table 2:** Pairwise comparison of different models using *cis*-enrichment and *trans*-enrichment

		FaST-LMM	geQTL-ridge	SET-eQTL	MTLasso2G	LORS	Matrix eQTL	Lasso
cis	geQTL ^+^	<0.0163	0.0124	<0.0001	<0.0001	<0.0001	<0.0001	<0.0001
	FaST-LMM	-	0.0247	<0.0001	<0.0001	<0.0001	<0.0001	<0.0001
	geQTL-ridge	-	-	<0.0001	<0.0001	<0.0001	<0.0001	<0.0001
	SET-eQTL	-	-	-	0.0117	<0.0001	<0.0001	<0.0001
	MTLasso2G	-	-	-	-	<0.0001	<0.0001	<0.0001
	LORS	-	-	-	-	-	<0.0001	0.0052
	Matrix eQTL	-	-	-	-	-	-	0.0134
		MTLasso2G	FaST-LMM	LORS	SET-eQTL	Matrix eQTL	Lasso	geQTL-ridge
trans	geQTL ^+^	0.0042	0.0040	0.0033	0.0029	0.0027	0.0022	0.0001
	MTLasso2G	-	0.0212	0.0134	0.0049	0.0042	0.0038	0.0005
	FaST-LMM	-	-	0.0233	0.0178	0.0125	0.0073	0.0006
	LORS	-	-	-	0.3110	0.1103	0.0151	0.0008
	SET-eQTL	-	-	-	-	0.1223	0.0578	0.0016
	Matrix eQTL	-	-	-	-	-	0.0672	0.0021
	Lasso	-	-	-	-	-	-	0.0025

#### Gene ontology enrichment analysis on detected group-wise associations for yeast

We further evaluate the quality of detected groups of genes by measuring the correlations between the detected groups of genes and the GO (Gene Ontology) categories [25]. Specifically, the GO enrichment test is calculated by DAVID [26]. In this paper, gene sets reported by our algorithm with calibrated *p*-values less than 0.01 are considered as significantly enriched.

**Fig. 6:**
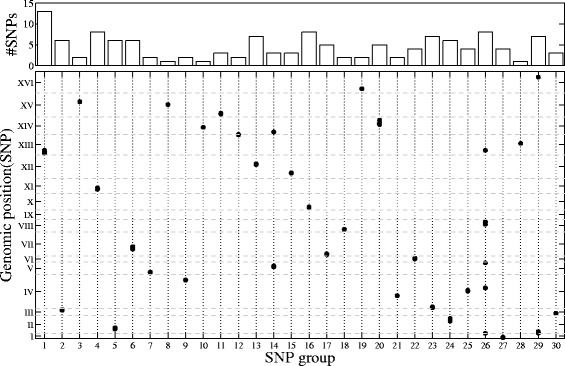
Genomic positions of SNPs in each SNP group

Since SET-eQTL is the only previous approach capable of detecting group-wise association mapping, we compare the groups of genes detected by geQTL and those by SET-eQTL. For SET-eQTL, 90 out of 150 gene sets are significantly enriched. By contrast, 28 out of 30 gene sets reported by geQTL are significantly enriched. This well illustrates that the effectiveness of geQTL to infer group-wise associations. The number of SNPs in each group reported by geQTL and their genomic locations are shown in Fig. 6. We can clearly observe that SNPs in the same group are often physically close to each other. This is reasonable because SNPs nearby usually jointly affect the expression level of a set of genes that achieves a specific cell function [8].

#### Reproducibility of eQTLs between studies

To further evaluate the identified associations, we investigate the consistency of calls between two independent studies [27]. We examine the reproducibility based on the following two criteria [14, 28, 29]:

- Reproducibility of detected SNP-gene associations: Let *L*_1_ and *L*_2_ be the sets of SNP-gene associations detected in the two yeast datasets, respectively. We can rank the associations according to the weights (or *q*-values for FaST-LMM). Let ${L_{1}^{T}}$ and ${L_{2}^{T}}$ be the top *T* most significant associations from the two datasets. The reproducibility is defined as $\frac {|{L_{1}^{T}}\bigcap {L_{2}^{T}}|}{T}$.
- Reproducibility of *trans* regulatory hotspots: For each SNP, we count the number of associated genes from the detected SNP-gene associations. We use this number as the *regulatory degree* of each SNP. For FaST-LMM, SNP-Gene pairs with a *q*-value < 0.001 are considered significant associations. We also tried different cutoffs for FaST-LMM (from 0.01 to 0.001), the results are similar. SNPs with large regulatory degrees are often referred to as hotspots. We sort SNPs in descending order of their regulatory degrees. We denote the sorted SNPs lists as *S*_1_ and *S*_2_ for the two yeast datasets. Let ${S_{1}^{T}}$ and ${S_{2}^{T}}$ be the top *T* SNPs in the sorted SNP lists. The trans calling consistency of reported hotspots is denoted by $\frac {|{S_{1}^{T}}\bigcap {S_{2}^{T}}|}{T}$.

**Fig. 7:**
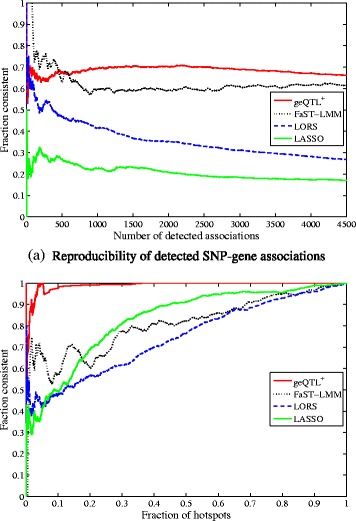
Reproducibility of eQTLs between two independent yeast eQTL datasets

In Fig. 7a, we show the consistency of the top 4500 associations between different studies. In Fig. 7b, we list the reproducibility of *trans* regulatory hotspots reported by different approaches. Overall, geQTL ^+^ yielded results with greater consistency all other methods. This well illustrates the superiority of geQTL ^+^.

## Conclusions

In literature, much efforts have been done on eQTL mapping. Traditional eQTL mapping approaches can not detect the group-wise associations between sets of SNPs and sets of genes. To achieve that, we propose a fast approach, *geQTL*, to detect both individual and group-wise associations for eQTL mapping. *geQTL* can also correct the effects of potential confounders. We also introduce efficient algorithms to scale up the computation so that the algorithms are able to tackle large scale data sets. Additionally, the proposed model provides an effective strategy to automatically infer the proper number of group-wise associations. We perform extensive experiments on both simulated datasets and yeast datasets to demonstrate the effectiveness and efficiency of the proposed method. Inferring individual and group-wise associations also helps us better explain the genetic basis of gene expression. Due to scalability issue, our model simply assume random errors between different genes are independent and have the same variance. That is the reason why current model only identified a small number of group-wise associations. Our future work will further incorporate the relationships between genes by integrating gene co-expression network or protein-protein-interaction network.

## Data availability

The software is publicly available at http://www.cs.unc.edu/~weicheng/bioinformatics_code.zip.

## Additional file

Additional file 1 Sparse regression models for unraveling group and individual associations in eQTL mapping. (PDF 1,004 KB)
