# Supplementary material for: Sparse regression models for unraveling group and individual associations in eQTL mapping
Source: BMC Bioinformatics. 2016 Mar 22;17:136. doi: 10.1186/s12859-016-0986-9 (PMC4802846; doi:10.1186/s12859-016-0986-9)
Supplement: Additional file 1 — Sparse regression models for unraveling group and individual associations in eQTL mapping. (PDF 1,004 KB) [file 12859_2016_986_MOESM1_ESM.pdf]

RESEARCH

Sparse regression models for unraveling group and individual associations in eQTL mapping

Wei Cheng[1], Yu Shi[2], Xiang Zhang[3] and Wei Wang[4*]
Emails:[1]weicheng@cs.unc.edu,[2]shiyu7500@gmail.com,[3]xiang.zhang@case.edu,[4]weiwang@cs.ucla.edu

[*]Correspondence: weiwang@cs.ucla.edu
[4]Department of Computer Science, University of California, Los Angeles, 3531-G Boelter Hall, CA 90095 Los Angeles, USA
Full list of author information is available at the end of the article

Keywords: eQTL mapping; group-wise association; computation efficiency

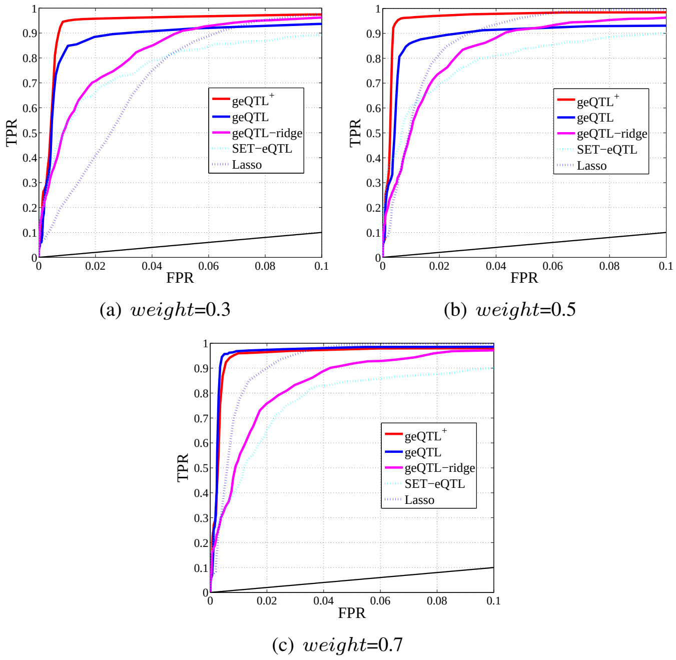

Figure 1 The ROC curve of FPR-TPR with different association weights. The black solid line denotes what random guessing would have achieved.

0.0.1 Performance Comparison on Simulated Data with Different Association Weights

Figure 1 shows the ROC curve of TPR-FPR for performance comparison with different association weights ($\mathcal{J} = 10, \tau = 0.1, SNR = 2$). The corresponding areas under the TPR-FPR curve are shown in Figure 2. It can be seen that geQTL and geQTL$^+$ outperform all alternative methods by a large margin since they consider both individual and group-wise associations. geQTL-ridge is not as good as geQTL and geQTL$^+$ because it does not provide a sparse solution for individual associations.

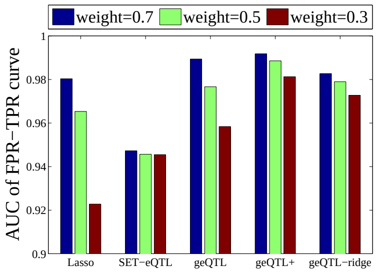

Figure 2 The areas under the precision-recall/FPR-TPR curve (AUCs) of different methods with different weights.

0.0.2 Performance Evaluation on Simulated Data for Detecting cis- and trans- Signals

To assess the ability of different methods to identify hidden features, we evaluate their performance on detecting cis- and trans- signals separately. Figure 3 shows the AUC curve of cis- and $trans$- signals when SNR=2.4. It can be seen that geQTL and geQTL$^+$ outperform geQTL-ridge for detecting both cis- and $trans$- signals. This is because the sparse feature selection method is more suitable for the association mapping problem. Figure 3 shows the performance of the proposed methods using different SNR's. Obviously, a larger SNR will obtain a better detection for both cis- and $trans$- signals.

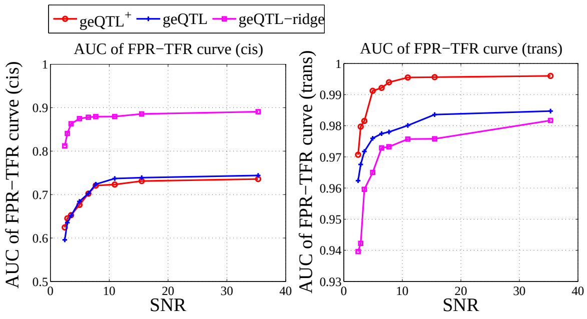

Figure 3 AUC of FPR-TFR curve(cis- and trans-) when varying the signal-to-noise ratios (SNR) on simulation dataset.

0.0.3 Computational Efficiency Evaluation

Scalability is an important issue for eQTL study. To evaluate the developed techniques for boosting the computational efficiency, we compare the running time of the methods that consider group-wise associations with/without these techniques. Figure 4 shows the running time when varying the number of SNPs K and number of traits N. LORS and Lasso are not considered here because they do not consider group-wise associations. We only measure the running time for N and K up to 900 in the figure so that we can compare geQTL and MTLasso2G which are time-consuming for optimization. We observe that MTLasso2G uses significantly more time than others. geQTL$^+$ uses less time than geQTL for excluding many unqualified elements in $\mathbf{C}$ in the optimization process. geQTL-ridge is more efficient than all other methods.

0.0.4 Shrinkage of $\mathbf{C}$ and $\mathbf{B} \times \mathbf{A}$

The group-wise associations are encoded in $\mathbf{B} \times \mathbf{A}$ and individual associations are encoded in $\mathbf{C}$. To enforce sparsity on $\mathbf{A}$, $\mathbf{B}$ and $\mathbf{C}$, we use ℓ_1 penalty on the elements of these matrices. Thus, it is interesting to study the the overall shrinkage of $\mathbf{B} \times \mathbf{A}$ and $\mathbf{C}$. We randomly generate 7 predictors ($\{\mathbf{x_1}, \mathbf{x_2}, ..., \mathbf{x_7}\}$) and 1 response ($\mathbf{z}$) with sample size 100. $\mathbf{x_i} \sim N(\mathbf{0}, 0.6 \cdot \mathbf{I})(i \in [1,7])$. The response vector was generated with the formula: $\mathbf{z} = 5 \cdot (\mathbf{x_1} + \mathbf{x_2}) - 3 \cdot (\mathbf{x_3} + \mathbf{x_4}) + 2 \cdot \mathbf{x_5} + \tilde{\epsilon}$ and $\tilde{\epsilon} \in N(\mathbf{0}, \mathbf{I})$. Thus, there are two groups of predictors ($\{\mathbf{x_1}, \mathbf{x_2}\}$ and $\{\mathbf{x_3}, \mathbf{x_4}\}$) and one individual predictor $\mathbf{x_5}$. Figure 5 shows the geQTL shrinkage of coefficients for $\mathbf{B} \times \mathbf{A}$ and $\mathbf{C}$ respectively. Each curve represents a coefficient as a function of the scaled parameter $s = \frac{|\mathbf{B} \times \mathbf{A}|}{\max |\mathbf{B} \times \mathbf{A}|}$ or $s = \frac{|\mathbf{C}|}{\max |\mathbf{C}|}$. We can see that the two groups of predictors can be identified by $\mathbf{B} \times \mathbf{A}$ as the most important variables, and the individual predictor can be identified by $\mathbf{C}$.

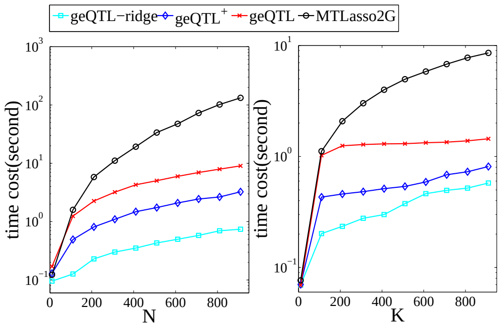

Figure 4 Running time performance when varying number of genes N and number of SNPs K.

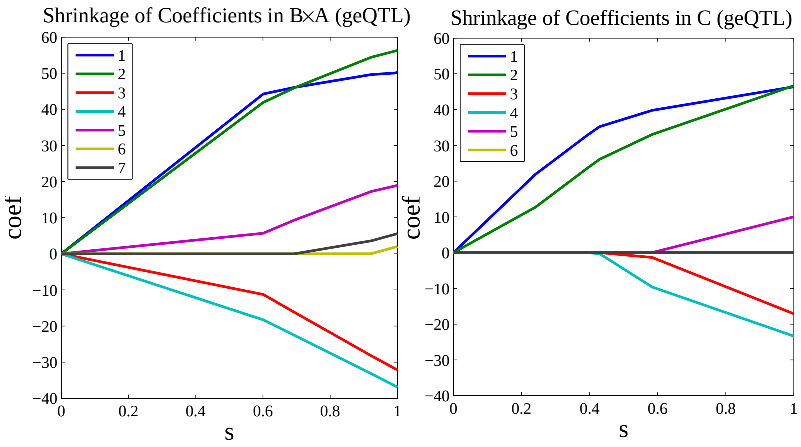

Figure 5 Model 2 shrinkage of coefficients for $\mathbf{B} \times \mathbf{A}$ and $\mathbf{C}$ respectively. Each curve represents a coefficient as a function of the scaled parameter $s = \frac{|\mathbf{B}\times\mathbf{A}|}{\max|\mathbf{B}\times\mathbf{A}|}$ or $s = \frac{|\mathbf{C}|}{\max|\mathbf{C}|}$.

0.0.5 Mouse eQTL Study

We use the genotypes of 184 partially inbred mice [1]. The original genotypes of 205,917 SNPs are collected using the mouse diversity array. The gene expression data are collected from the liver. After removing the genes containing missing values, we obtain 9947 gene profiles. Since no baseline method can handle dataset at this scale, we first use Matrix eQTL [1] [2] to find the SNPs that have at least one eQTL with FDR$< 10^{-10}$. This step selects 10534 SNPs. Only geQTL$^+$, geQTL-ridge and Lasso can process this filtered dataset. It takes 40 minutes for geQTL-ridge, around 1 hour for geQTL$^+$, and 3 days for Lasso to run to completion. geQTL$^+$ reports stronger *cis*-regulatory signals and weaker *trans*-regulatory bands than Lasso. The top-10000 association signals detected by Lasso and geQTL$^+$ are shown in Figure 6. We can see clearly that geQTL$^+$ reports stronger *cis*-regulatory signals and weaker *trans*-regulatory bands than Lasso.

0.1 Setting Proper Number of Group-Wise Associations M

The singular values of $(\mathbf{Z} - \mathbf{L} - \mathbf{C}\mathbf{X})\mathbf{X}^T(\mathbf{X}\mathbf{X}^T)^{-1}$ for each data set are shown in Figure 7. The dash line indicates value of M. We can draw a plot with singular values of $(\mathbf{Z} - \mathbf{L} - \mathbf{C}\mathbf{X})\mathbf{X}^{\mathrm{T}}(\mathbf{X}\mathbf{X}^{\mathrm{T}})^{-1}$ in descending order and set M to be k, if the first k singular values are large and significantly greater than the $(k+1)$-th singular value. To achieve this, we can calculate all the gaps of successive singular values and locate the maximal gap.

Author details

[1]Department of Computer Science, UNC at Chapel Hill, 201 S Columbia St., NC 27599 Chapel Hill, USA. [2]Computer Science at the University of Illinois at Urbana-Champaign, 201 North Goodwin Avenue, IL 61801 Urbana, USA. [3]Department of Elect. Eng. and Computer Science, Case Western Reserve University, 10900 Euclid Avenue, OH 44106 Cleveland, USA. [4]Department of Computer Science, University of California, Los Angeles, 3531-G Boelter Hall, CA 90095 Los Angeles, USA.

[1]The software is publicly available at http://www.bios.unc.edu/research/genomic_software/Matrix_eQTL/

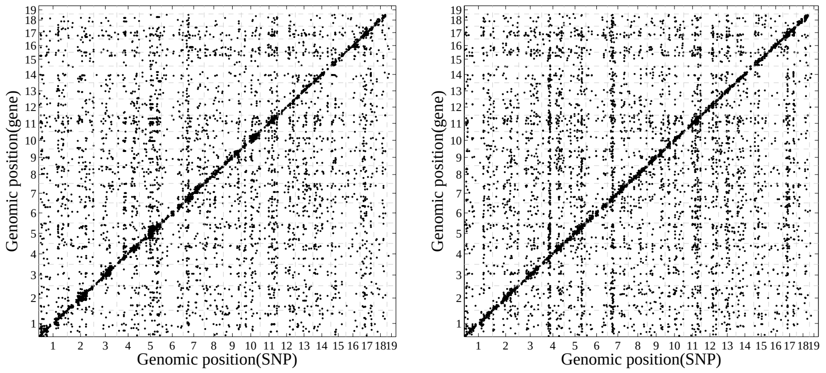

(a) geQTL^{+} (b) Lasso

Figure 6 Significant associations discovered by different methods in mouse data. The top 10000 associations ranked by abs(weight) are shown in each plot unless otherwise noted. The x-axis represents SNPs and y-axis represents genes (traits). Both SNPs and genes are arranged by their locations in the genome. geQTL^{+} has stronger *cis*-regulatory signals and weaker *trans*-regulatory bands than Lasso.

References

1. Aylor, D.L., Valdar, W., et al.: Genetic analysis of complex traits in the emerging Collaborative Cross. Genome Res. **21**(8), 1213–1222 (2011)
2. Shabalin, A.A.: Matrix eQTL: ultra fast eQTL analysis via large matrix operations. Bioinformatics **28**(10), 1353–1358 (2012)

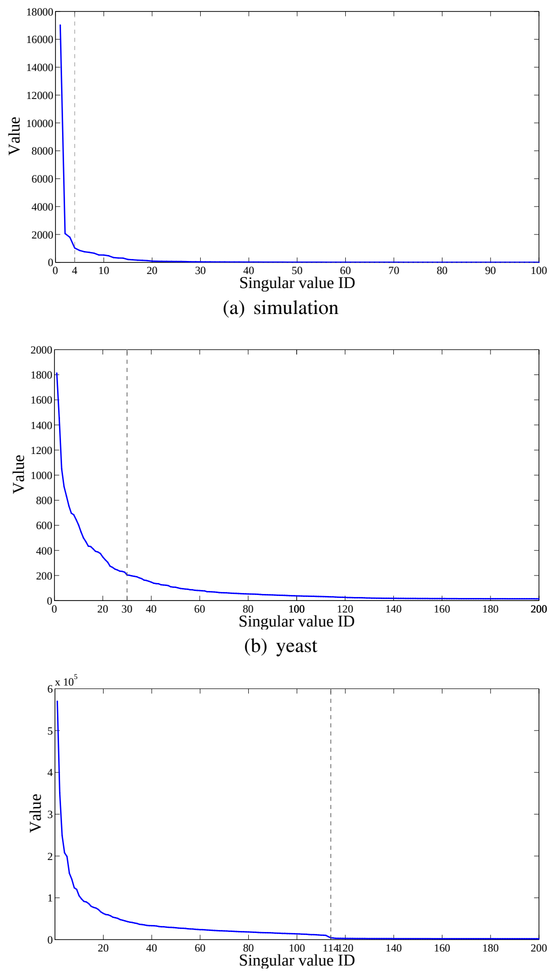

(a) simulation

(b) yeast

(c) mouse

Figure 7 Singular values of $(\mathbf{Z} - \mathbf{L} - \mathbf{CX})\mathbf{X}^T(\mathbf{XX}^T)^{-1}$. For yeast and mouse data, we have trucated the x-axis to 200 for clarity.
